# Stress corrosion cracking behavior of 20MnTiB high-strength bolts in simulation of humid climate in Chongqing

**DOI:** 10.1038/s41598-021-03302-y

**Published:** 2021-12-13

**Authors:** Lin Chen, Juan Wen, Luyu Zhang, Zheng Li, Guangwen Li, Chengwu Ming

**Affiliations:** 1 Yibin University, Yibin, 644000 People’s Republic of China; 2 Chongqing Cheng Tou Road and Bridge Administration Co. Ltd, Chongqing, 400060 People’s Republic of China; 3 School of New Energy and Materials, Southwest Petroleum University, Chengdu, 610500 People’s Republic of China

**Keywords:** Civil engineering, Structural materials, Environmental impact

## Abstract

20MnTiB steel is the most widely used high-strength bolt material for steel structure bridges in China, and its performance is of great significance to the safe operation of bridges. Based on the investigation of the atmospheric environment in Chongqing in recent years, the corrosion solution to simulate the humid climate of Chongqing was designed in this study, and the stress corrosion experiment of high-strength bolts in the simulated humid climate of Chongqing was carried out. The effects of temperature, pH and concentration of simulated corrosion solution on the stress corrosion behavior of 20MnTiB high-strength bolts were studied.

## Introduction

20MnTiB steel is the most widely used high-strength bolt material for steel structure bridges in China, and its performance is of great significance to the safe operation of bridges. Li et al.^1^ tested the properties of 20MnTiB steel commonly used for grade 10.9 high-strength bolts at high temperature in the range of 20–700 °C, and obtained the stress–strain curve, yield strength, tensile strength, Young`s modulus, elongation and expansion coefficient. Zhang et al.^2^, Hu et al.^3^ etc., analyzed the fracture of 20MnTiB high-strength bolts used in steel bridge through chemical compositions examination, mechanical properties test, microstructure examination, the macro and micro analysis of the thread tooth surface and fracture surface, and the results show that the main reason of high-strength bolt fracture is related to the thread defect,which generates large stress concentration, and both stress concentration of crack tip and corrosion condition in the open air cause the stress corrosion cracking.

High-strength bolts used in steel bridge usually serviced in humid atmosphere for a long time. Factors as high humidity, high temperature and the settlement and absorption of harmful substances in the environment are very easy to cause corrosion of the steel structure. Corrosion will lead to section loss of high-strength bolts, produce lots of defects and cracks. And these defects and cracks will continue to expand, so as to reduce the life of high-strength bolts and even led to its fracture. So far there are many researches on the influence of environmental corrosion on the stress corrosion properties of materials. Catar et al.^4^ studied the stress corrosion behavior of magnesium alloy with different aluminum content in acid, alkaline and neutral environment by slow strain rate test (SSRT). Abdel et al.^5^ studied the electrochemical and stress corrosion cracking behavior of Cu10Ni alloy in 3.5% NaCl solution in the presence of different concentrations of sulfide ions. Aghion et al.^6^ evaluated the corrosion performance of die casting magnesium alloy MRI230D in 3.5% NaCl solution by immersion test, salt spray test, potentiodynamic polarization analysis and SSRT. Zhang et al.^7^ studied the stress corrosion behavior of 9Cr martensitic steel by means of SSRT and traditional electrochemical testing technology, and obtained the influence rule of chloride ion on the static corrosion behavior of martensitic steel at room temperature. Chen et al.^8^ studied the stress corrosion behavior and cracking mechanism of X70 Steel in the simulated solution of sea mud containing SRB at different temperatures by SSRT. Liu et al.^9^ studied the effect of temperature and tensile strain rate on the seawater stress corrosion resistance of 00Cr21Ni14Mn5Mo2N austenitic stainless steel by means of SSRT. The results showed that the temperature in the range of 35–65 °C had no significant effect on the stress corrosion behavior of the stainless steel. Lu et al.^10^ evaluated the delayed fracture sensitivity of samples with different tensile strength grades through the constant load delayed fracture test and SSRT. It is suggested that the tensile strength of 20MnTiB steel and 35VB steel high-strength bolts should be controlled at 1040–1190 mpa. However, most of these studies are basically to use a simple 3.5% NaCl solution to simulate the corrosion environment, while the actual service environment of the high-strength bolts is more complex, and there are many influencing factors, such as the pH value of the solution, temperature, concentration of corrosion medium, etc. Ananya et al.^11^ studied the effect of environmental parameters and the material in corrosion medium on the corrosion and stress corrosion cracking of duplex stainless steel. Sunada et al.^12^ carried out the stress corrosion cracking test of SUS304 steel at room temperature in aqueous solution containing H_2_SO_4_ (0–5.5 kmol/m^−3^) and NaCl (0–4.5 kmol/m^−3^). And the effect of H_2_SO_4_ and NaCl on corrosion type of SUS304 steel was studied. Merwe et al.^13^ studied the effect of rolling direction, temperature, CO_2_/CO concentration, air pressure and corrosion time on the stress corrosion sensitivity of A516 pressure vessel steel by means of SSRT. Ibrahim et al.^14^ used NS4 solution as groundwater simulation solution to study the effect of environmental parameters such as concentration of bicarbonate ion (HCO), pH and temperature on stress corrosion cracking of API-X100 pipeline steel after peeling off the coating. Shan et al.^15^ studied the change rule of stress corrosion cracking sensitivity of austenitic stainless steel 00Cr18Ni10 with temperature under the black water medium condition of simulated coal hydrogen plant through SSRT under different temperature conditions (30–250°C). Han et al.^16^ characterized the hydrogen embrittlement sensitivity of high-strength bolt samples by using constant load delayed fracture test and SSRT. Zhao^17^ studied the effect of pH, SO_4_^2−^, Cl^−1^ on the stress corrosion behavior of GH4080A alloy by means of SSRT. The results showed that the lower the pH, the worse the stress corrosion resistance of GH4080A alloy. It has obvious stress corrosion sensitivity to Cl^−1^, and is not sensitive to SO_4_^2−^ ion medium at room temperature. However, there are few studies on the influence of environmental corrosion on 20MnTiB steel high-strength bolts.

To figure out the reasons for the failure of high-strength bolts used in bridges, the author has carried out a series of studies.First,the spatial and temporal distribution characteristics of failed high strength bolts in the Chaotianmeng Bridge were analyzed^18^, then representative failed high strength bolts samples were selected and the causes of the failure of these samples were discussed from the perspectives of chemical composition, micromorphology of fractures, metallographic structure and mechanical property analysis^19,20^. Based on the investigation of the atmospheric environment in Chongqing in recent years, the corrosion solution to simulate the humid climate of Chongqing was designed. The stress corrosion experiment, the electrochemical corrosion experiment and the corrosion fatigue experiment of high-strength bolts in the simulated humid climate of Chongqing were carried out. In this study, the effects of temperature, pH and concentration of simulated corrosion solution on the stress corrosion behavior of 20MnTiB high-strength bolts were studied through mechanical properties test, the macro and micro analysis of the fracture surface and the surface corrosion products.

## Experimental

### Simulation of humid climate in Chongqing

Chongqing is located in Southwest China and the upper reaches of the Yangtze River, which belongs to a subtropical monsoon humid climate. The annual average temperature is 16–18 °C, the annual average relative humidity is mostly 70–80%, the annual sunshine hours are 1000–1400 h, and the sunshine percentage is only 25–35%.

According to relevant reports on sunshine and ambient temperature of Chongqing from 2015 to 2018, the daily average temperature of Chongqing is 17 °C at the lowest and 23 °C at the highest, and the maximum temperature of Chaotianmen Bridge body in Chongqing can reach 50 °C^21,22^. Thus the temperature level of stress corrosion test was set at 25 °C and 50 °C.

The pH value of simulatedcorrosion solution directly determines the amount of H^+^, but it is not simply that the lower the pH value is, the more likely corrosion will occur. The effects of pH valueon the results will be different for different materials and solutions. In order to better study the effect of simulated corrosionsolution on stress corrosion performance of high-strength bolts, the pH value of stress corrosion experiment is set as 3.5, 5.5 and 7.5, in combination with literature research^23^ and the annual rainwater pH range of Chongqing from 2010 to 2018.

The higher the concentration of simulated corrosion solution is, the more the ion content in the simulated corrosion solution is, the more the effect on the performance of the material is. In order to study the effect of the concentration of simulated corrosion solution on the stress corrosion of high-strength bolts, and to realize the accelerated corrosion test in the artificial laboratory, the concentration of simulated corrosion solution was set as 4 levels, no corrosion, original simulatedcorrosion solution concentration (1 ×), 20 × the original simulated corrosion solution concentration (20 ×) and 200 × the original simulated corrosion solutionconcentration (200 ×), respectively.

The environment that of the temperature of 25 °C, pH of 5.5, and the concentration of the original simulated corrosion solution is closest to that of the actual service condition of the high-strength bolt used in bridge. However, to speed up the corrosion test process, the experimental conditions of the temperature of 25 °C, pH of 5.5 and 200 × the concentration of the original simulated corrosion solution was set as the reference control group. When the effects of temperature, concentration or pH of the simulated corrosion solution on the stress corrosion performance of the high-strength bolt are examined respectively, other factors remained unchanged as the experimental level of the reference control group.

#### Preparation of corrosion solution for simulating humid climate environment in Chongqing

According to the brief report on atmospheric environment quality issued by Chongqing Municipal Bureau of ecological environment in 2010–2018, and referring to the precipitation composition in Chongqing reported Zhang^24^ and other literature, the simulated corrosion solution was designed by promoting the concentration of SO_4_^2−^ based on the precipitation composition in the main urban area of Chongqing in 2017. The composition of the simulated corrosion solution is shown in Table 1 as below:

**Table 1:** Corrosion solution composition of simulated precipitation in Chongqing(mg/L).

Composition	SO_4_^2−^	NO_3_^−^	NH_4_^+^	Ca^2+^	Mg^2+^	Cl^−^	K^+^	Na^+^	F^−^
Simulated corrosion solution (1x)	10.24	6.57	3.77	3.82	0.19	0.59	0.5	0.21	0.11
2017 (Hai fu)	8.39	6.57	3.77	3.82	0.19	0.59	0.5	0.21	0.11

The simulated corrosion solution was prepared with analytical reagent and distilled water according to the chemical ion concentration balance method. And the pH value of simulated corrosion solution was adjusted by using the precision pH meter, nitric acid solution and sodium hydroxide solution.

#### Experimental device for simulating humid climate environment in Chongqing

**Figure 1:**
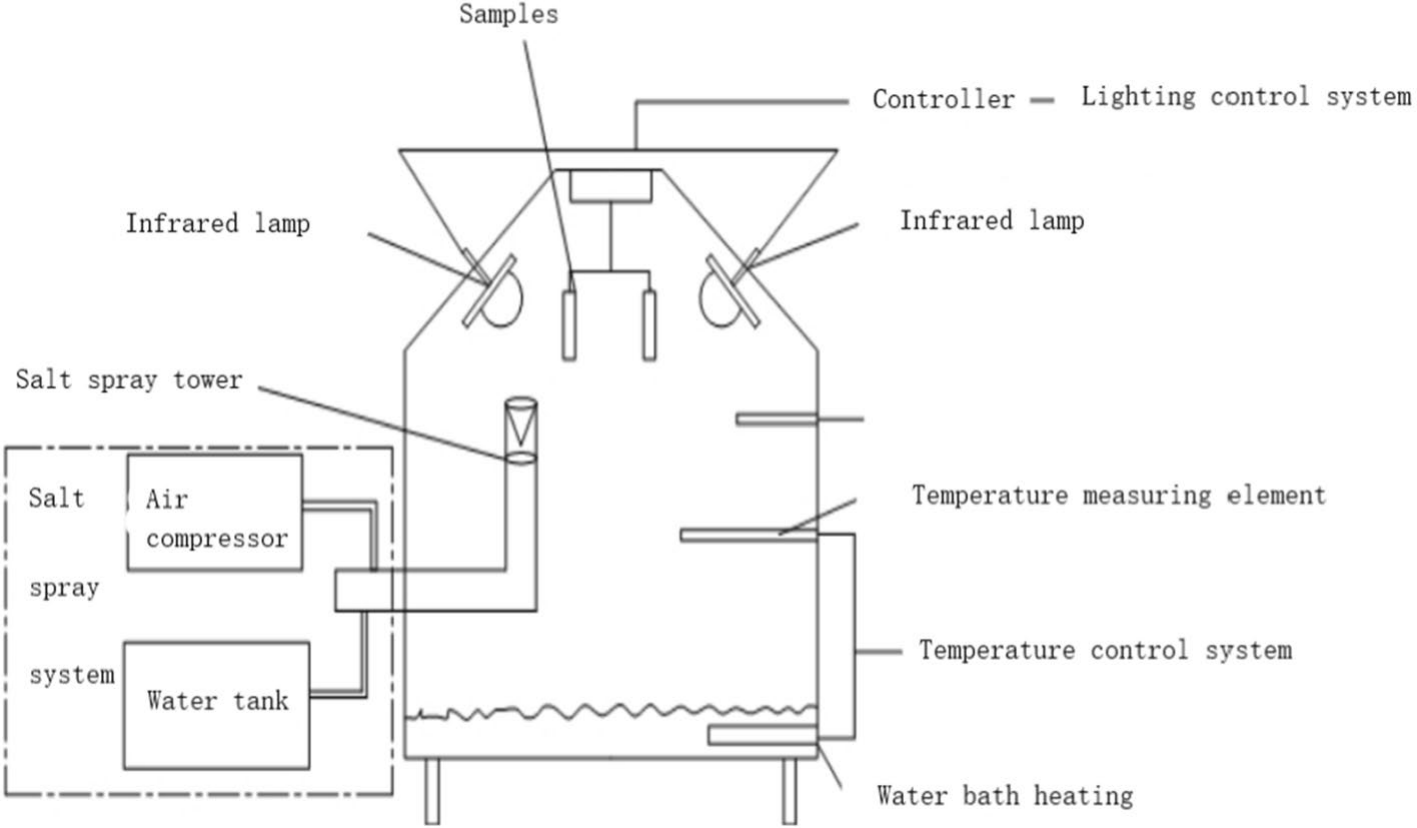
Schematic diagram of salt spray test device simulating atmospheric corrosion.

In order to simulate the humid climate environment in Chongqing, the salt spray tester was specially modified and designed^25^. As shown in Fig. 1, there are two systems in this experimental equipment: the salt spray system and the light system. Salt spray system is the main function of this experimental equipment, which is composed of control part, spray part and induction part. The function of the spray part is to pump salt spray into the test chamber by air compressor. The induction part is composed of temperature measuring element, which induct the temperature in the test chamber. The control part is composed of microcomputer, which connects spray part and induction part, controlling the whole experiment process. The lighting system is installed in the salt spray test chamber to simulate sunshine. The lighting system is composed of infrared lamp and time controller. At the same time, a temperature sensor is installed in the salt spray test chamber for real-time monitoring of the temperature around the sample.

### Stress corrosion test method

The stress corrosion samples under constant load were processed according to NACETM0177-2005 standard (Laboratory Testing of Metals for Resistance to Sulfide Stress Cracking and Stress Corrosion Cracking in H_2_S Environments). The stress corrosion samples were first cleaned by acetone and ultrasonic mechanical cleaning to remove the oil residual, and then dehydrated with alcohol and dried in a drying oven. Then the clean samples were placed in the test chamber of the salt spray test device to simulate the corrosion under humid climate environment of Chongqing. According to the standard NACETM0177-2005 and the salt spray test standard GB/T 10,125–2012, the experimental time of stress corrosion under constant load in this study was uniformly determined to be 168 h. The tensile test of the corroded samples under different corrosion conditions was carried out on the MTS-810 universal tensile testing machine, and the mechanical properties and fracture corrosion morphology were analyzed.

## Results

### Analysis of corrosion morphology

**Figure 2:**
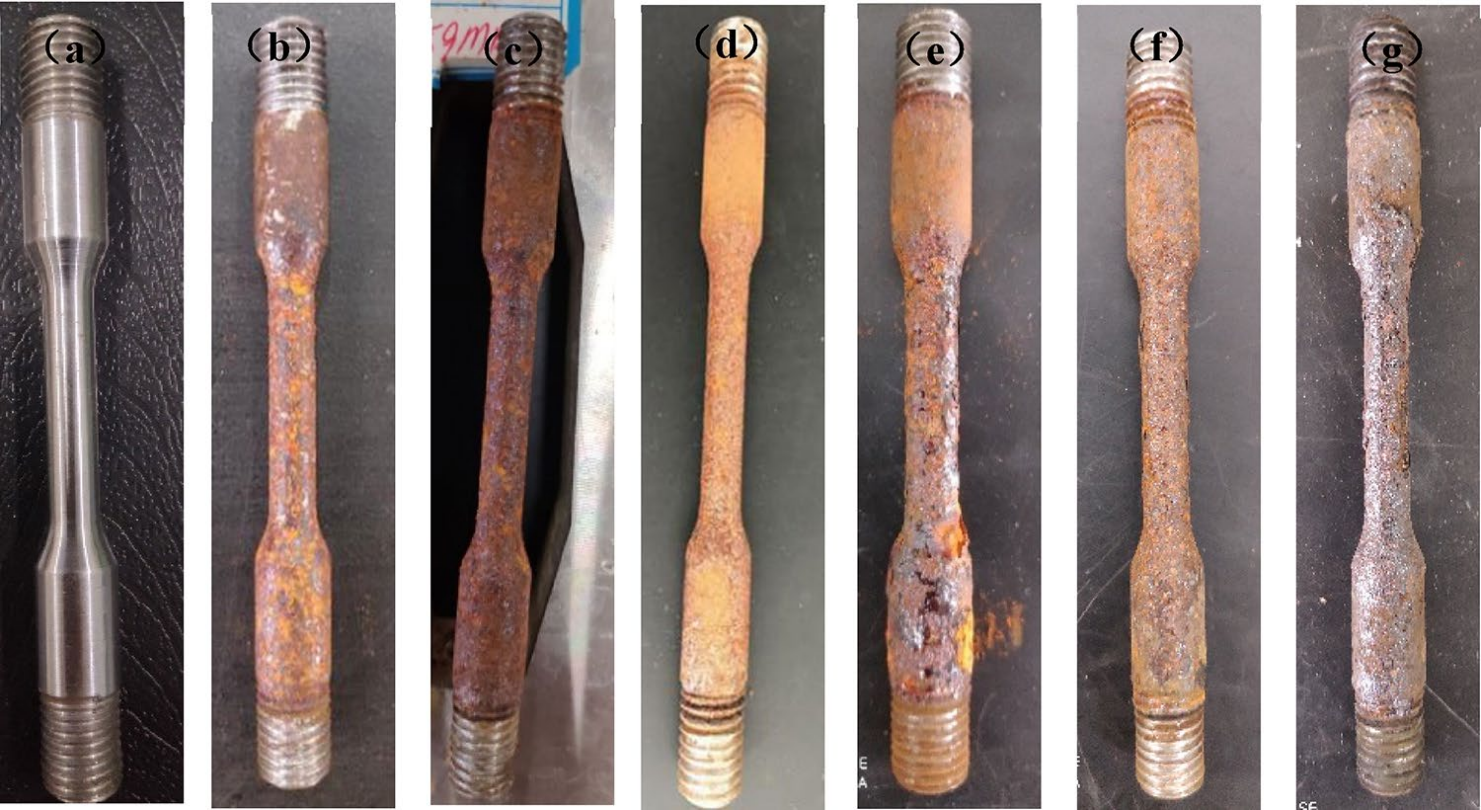
Macro-morphology of stress corrosion samples of 20MnTiB high-strength bolts in different simulated corrosion environments: (**a**) non-corrosion; (**b**) 1 time; (**c**) 20 ×; (**d**) 200 ×; (**e**) pH3.5; (**f**) pH7.5; (**g**) 50 °C.

**Figure 3:**
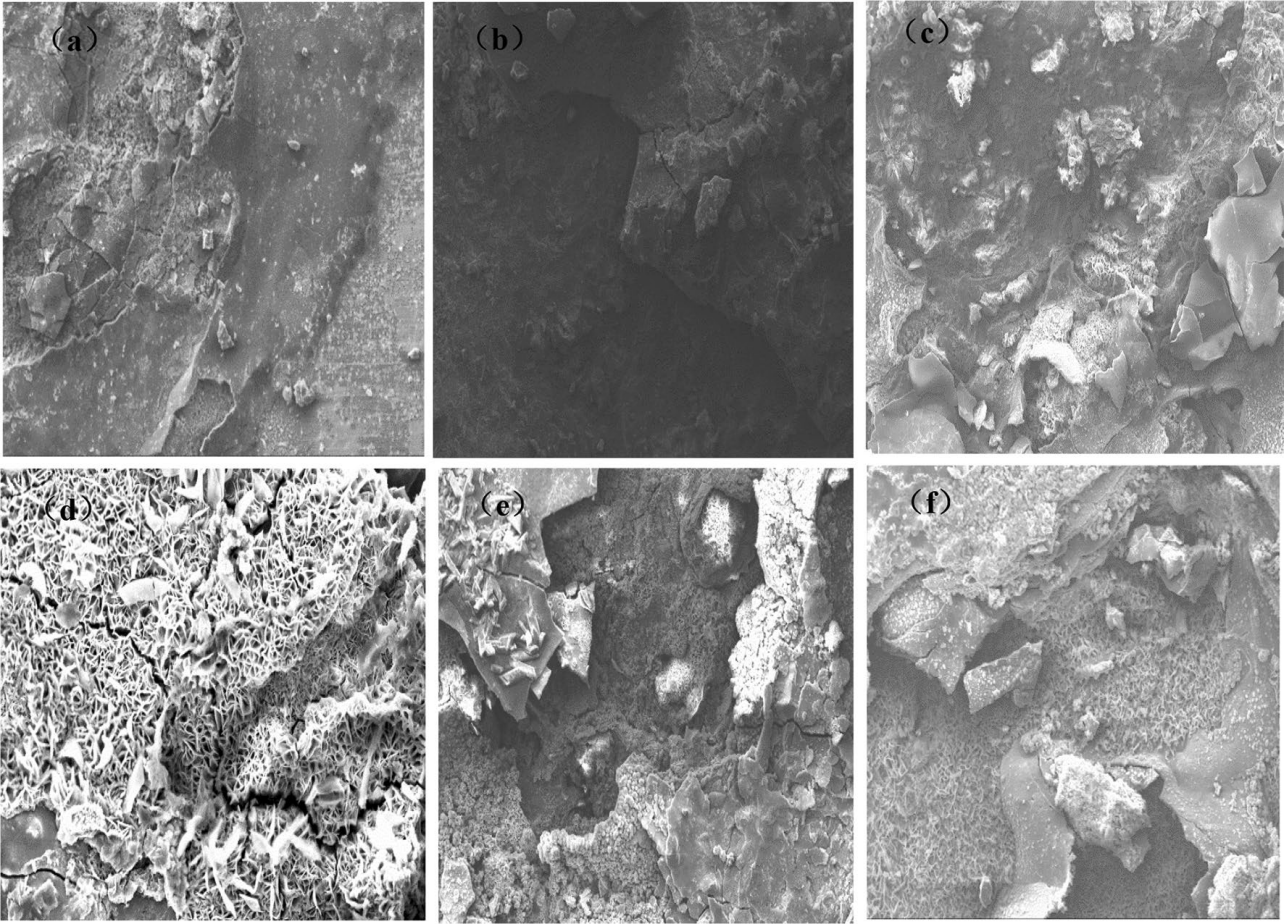
Micro morphology of corrosion products of 20MnTiB high-strength bolts in different simulated corrosion environments (100 ×): (**a**)1 time; (**b**) 20 ×; (**c**) 200 ×; (**d**) pH3.5; (**e**) pH7.5; (**f**) 50 °C.

The macro morphology and micro morphology of the surface corrosion of stress corrosion samples of high-strength bolts under different corrosion conditions are shown in Figs. 2 and 3 respectively.

It can be seen from Fig. 2a that the surface of the high-strength bolt sample without corrosion shows bright metallic luster and no obvious corrosion phenomenon. While under the condition of the original simulated corrosion solution (Fig. 2b), there are sepia and brownish red corrosion products partially covering the sample surface, and some areas of the surface still show obvious metallic luster, which indicates that only some areas of the sample surface have pitting corrosion, and the simulated corrosionsolution has little impact on the performance of the material. But under the condition of 20 × the concentration of the original simulated corrosion solution (Fig. 2c), the surface of the high-strength bolt sample has been completely covered by a large number of sepia corrosion products and a small number of brownish red corrosion products, no obvious metal luster is found, and there is a few brownish black corrosion products near the substrate surface. And under the condition of 200 × the concentration of the original simulated corrosion solution (Fig. 2d), the surface of the sample is completely covered by sepia corrosion products, and some areas appear brownishblack corrosion products.

With the decrease of pH value to 3.5 (Fig. 2e), the sepia corrosion products on the surface of the sample are the most, and some corrosion products have peeled off.

Figure 2g shows that with the increase of temperature to 50 °C, the content of brownish red corrosion products on the surface of the sample decreases sharply, while the bright sepia corrosion products cover the surface of the sample on a large scale. The corrosion products layers are relatively loose, and some brownish black products have peeled off.

As shown in Fig. 3, in different corrosion environments, the corrosion products on the surface of 20MnTiB high-strength bolt stress corrosion samples are obviously stratified, and the thickness of the corrosion layer increases with the concentration of simulated corrosionsolution. Under the condition of the original simulated corrosionsolution (Fig. 3a), the corrosion products on the sample surface can be divided into two layers: the corrosion products on the outermost layer are evenly distributed, but a large number of turtle cracks appear; the inner layer is loose cluster corrosion products. Under thecondition of 20 × the concentration of the original simulated corrosion solution (Fig. 3b), the corrosion layer on the sample surface can be divided into three layers: the outermost layer is mainly scattered cluster corrosion products, which are loose and porous and do not have good protection performance; the middle layer is a uniform corrosion product layer, but there are obvious cracks, so that the corrosive ions can pass through the cracks and erode the matrix; The inner layer is a dense corrosion product layer without obvious cracks, which has a good protective effect on the matrix. Under the condition of 200 × the concentration of the original simulated corrosion solution (Fig. 3c), the corrosion layer on the sample surface can be divided into three layers: the outermost layer is a thin and uniform corrosion product layer; the middle layer is mainly petal shaped and sheet-like corrosion products; and the inner layer is a dense corrosion product layer without obvious cracks and holes, which has a good protective effect on the matrix.

It can be seen from Fig. 3d that in the simulated corrosion environment of pH 3.5, there are a large number of flocculent or acicular corrosion products on the surface of 20MnTiB high-strength bolt samples. It is speculated that these corrosion products are mainly γ- FeOOH and a small amount of α- FeOOH staggered distribution^26^, and there are obvious cracks in the corrosion layer.

It can be seen from Fig. 3f that when the temperature increased to 50 °C, no obvious dense inner rust layer is found in the structure of corrosion layer, indicating that there is a gap between the corrosion layers at 50 °C, which makes the matrix not completely covered by corrosion products to supply protection, and the corrosion tendency of the matrix is deepened.

### Loss analysis of mechanical properties

The mechanical properties of high-strength bolts under constant load stress corrosion in different corrosion environments are shown in Table 2:

**Table 2:** Mechanical properties of 20MnTiB high-strength bolt sample under constant load stress corrosion in different simulated corrosion environments (ϕ = 6.35 mm).

Condition	Yield strength/MPa	Tensile strength/MPa	Elongation/%	Section shrinkage/%
Non-corrosion	1116	1228	17.48	44.88
1time concentration	1143	1215	17.78	43.78
20 × concentration	1126	1188	16.34	43.15
**200 ×** concentration	**1148**	**1191**	**16.31**	43.62
pH3.5	1115	1176	16.05	45.35
pH7.5	1139	1193	15.27	43.15
50°C	1130	1186	15.77	42.05
Standard ≥	940	1140	12	42

It can be seen from Table 2 that the mechanical properties of 20MnTiB high-strength bolt samples still meet the standard requirements after the dry and wet cycle accelerated corrosion test under different simulated corrosion environments, but there are some damages occur compared with the non-corroded sample. Under the concentration of the original simulated corrosion solution, the mechanical properties of the sample did not change significantly, but under the concentration of 20 × or 200 × of the simulated solution, the elongation of the sample decreased significantly. The mechanical properties were similar under 20 × and 200 × the concentration of the original simulated corrosion solution. When the pH of the simulated corrosion solution decreased to 3.5, the tensile strength and elongation of the sample decreased significantly. When the temperature rises to 50°C, the tensile strength and elongation decrease significantly, and the shrinkage of the section is very close to the standard value.

### Macroscopic and microscopic analysis of fracture

**Figure4:**
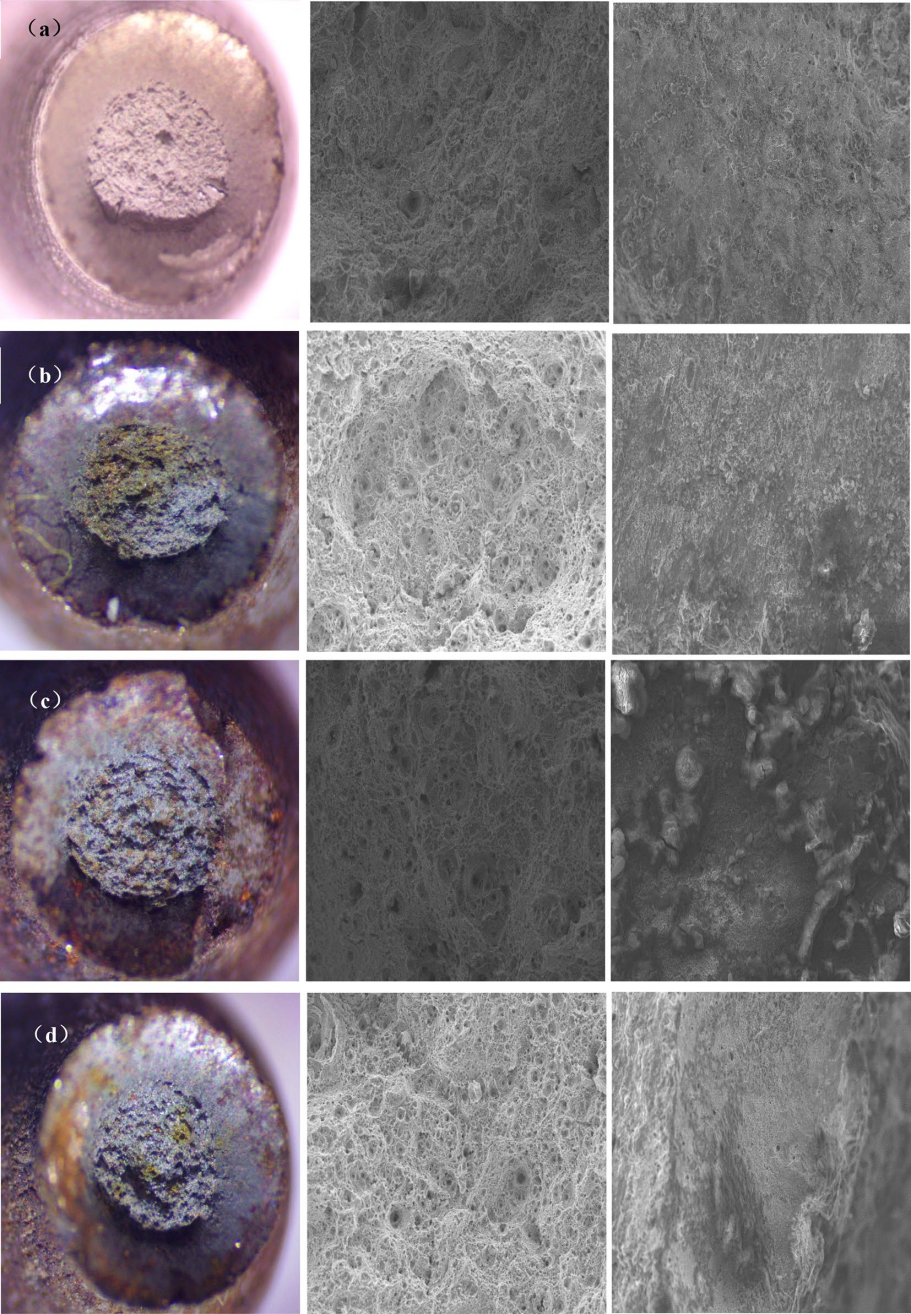

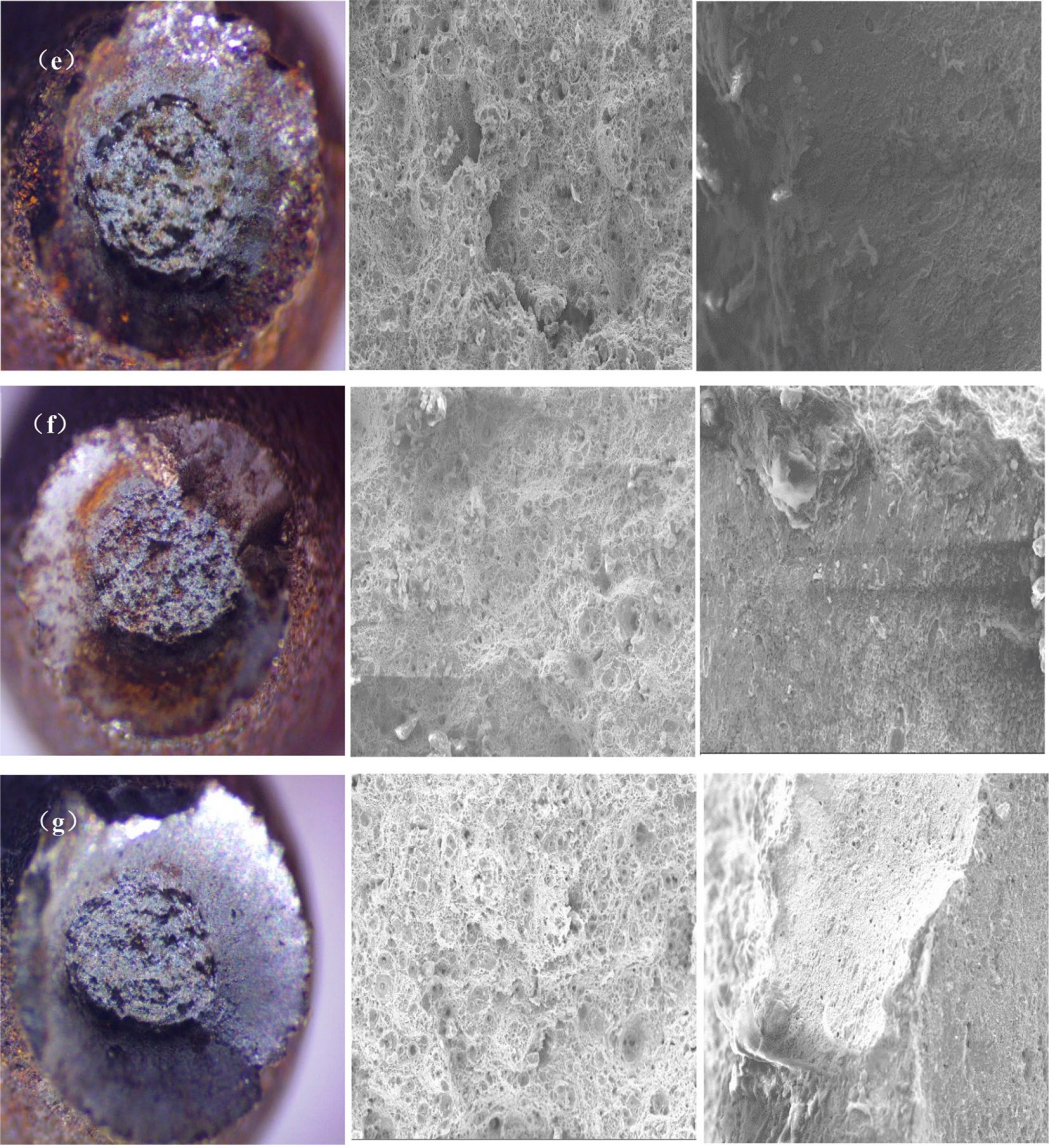
Macro and micro fracture morphology of 20MnTiB high-strength bolt sample in different simulated corrosion environments (500 ×): (**a**) non-corrosion; (**b**) 1 time; (**c**) 20 ×; (**d**) 200 ×; (**e**) pH3.5; (**f**) pH7.5; (**g**) 50 °C.

The fracture morphology of 20MnTiB high-strength bolt stress corrosion samples under different corrosion environments is shown in Fig. 4, in turn is the macro morphology of the fracture, the central fiber area of the fracture, the micro morphology of the interface between the shear lip edge and the sample surface.

It can be seen from Fig. 4 that the fracture of stress corrosion samples of 20MnTiB high-strength bolts in different simulated corrosion environments presents a typical cup cone fracture, and compared with the samples without corrosion (Fig. 4a), the area of fiber area in the center of fracture is relatively smaller, and the area of shear lip area is larger. This shows that after corrosion, the mechanical properties of the material are damaged obviously. With the increase of the concentration of the simulated corrosion solution, the dimple in the central fiber area of the fracture increased, and obvious tearing slitsappeared. When the concentration increases to 20 × of the original simulated corrosion solution concentration, there are obvious corrosion pits at the interface between the edge of the shear lip and the surface of the sample, and there are a lot of corrosion products on the surface of the sample.

As deduced in Fig. 3d that the corrosion layer on the sample surface has obvious cracks, and does not provide good protection for the matrix, in the simulated corrosion solution of pH3.5 (Fig. 4e), the sample surface is serious corroded, and the central fiber area is obviously small, and there are a lot of irregular tearing slits in the center of the fiber area. With the increase of the pH value of the simulated corrosion solution, the tearing band of the central fiber area of the fracture surface decreased, the dimple gradually decreased, and the dimple depth also gradually decreased.

When the temperature increased to 50 °C (Fig. 4g), the shear lip aere of the fracture surface of the sample is the largest, the dimple in the central fiber area is significantly increased, and the dimple depth is also increased, and the corrosion products and pits at the interface between the edge of the shear lip and the sample surface are increased, which confirms the deepening corrosion tendency of matrix reflected in Fig. 3f.

## Discussion

### Influence of pH value of corrosion solution

The pH value of corrosion solution will cause certain damage to the mechanical properties of 20MnTiB high-strength bolt, but the effect is not significant. In the corrosion solution of pH 3.5, a large number of flocculent or acicular corrosion products are distributed on the surface of the sample, and there are obvious cracks in the corrosion layer, which can not form a good protection for the matrix. And there are significant corrosion pits and a large number of corrosion products in the micro morphology of the sample fracture. This shows that the ability of the samples to resist the external force deformation decreases significantly in the acid environment, and the degree of stress corrosion tendency of the material increases significantly.

### Influence of concentration ofcorrosion solution

The original simulated corrosion solution has little effect on the mechanical properties of the high-strength bolt samples, but with the concentration of the simulated corrosion solution increasing to 20 × of the original simulated corrosion solution concentration, the mechanical properties of the samples are obviously damaged, and there are significant corrosion pits, secondary cracks and a large number of corrosion products in the fracture micro morphology. When the concentration of the simulated corrosion solution increases from 20 to 200 × the concentration of the original simulated corrosion solution, the effect of the concentration of the corrosion solution on the mechanical properties of the material is weakened.

### Influence of temperature

The yield strength and tensile strength of 20MnTiB high-strength bolt samples changed little compared with those of non corroded samples when the simulated corrosion temperature was 25 °C. While under the simulated corrosion environment temperature of 50 °C, the tensile strength and elongation of the sample decreased significantly, the section shrinkage was close to the standard value, the shear lip of the fracture was the largest, the dimple in the central fiber area increased significantly and the dimple depth increased, and the corrosion products and corrosion pits increased. This indicated that the temperature synergistic corrosion environment had a great impact on the mechanical properties of high-strength bolts, which is not obvious at room temperature, but more significant when the temperature reaches 50 °C.

## Conclusion

After the indoor accelerated corrosion test simulating Chongqing atmospheric environment, the tensile strength, yield strength, elongation and other parameters of 20MnTiB high-strength bolt have been reduced, and obvious stress damage has occurred. As the material is in the state of stress, obvious local corrosion acceleration occur. And because of the comprehensive effect of stress concentration and corrosion pit, it is easy to cause obvious plastic damage of high-strength bolt, and reduce the ability to resist external force deformation, and increase the tendency of stress corrosion.
